# Menopausal symptoms assessment among middle age women in Kushtia, Bangladesh

**DOI:** 10.1186/1756-0500-4-188

**Published:** 2011-06-15

**Authors:** Shahedur Rahman, Faizus Salehin, Asif Iqbal

**Affiliations:** 1 Department of Biotechnology and Genetic Engineering, Islamic University, Kushtia-7003, Bangladesh; 2 Department of Biotechnology and Genetic Engineering, University of Development Alternative, Dhaka, Bangladesh

## Abstract

**Background:**

There are few menopausal study data available in South East Asia especially in Bangladesh. This study was conducted in a well populated town named Kushtia, which is located in the western part of Bangladesh.

**Objectives:**

This study was aimed to document the menopausal-related symptoms among middle age women of Kushtia region of Bangladesh.

**Methods:**

By using modified MRS (Menopause Rating Scale) questionnaire, 509 women aged 40-70 years were interviewed to document symptoms commonly associated with menopause.

**Findings:**

The mean age of menopause was 51.14 years. The most prevalent symptoms reported include, feeling tired (92.90%); headache (88.80%); joint and muscular discomfort (76.20%); physical and mental exhaustion (60.90%) and sleeplessness (54.40%) which are followed by depressive mood (37.30%); irritability (36%); dryness of vagina (36%); hot flushes and sweating (35.80%); anxiety (34.20%). However, noted less frequent symptoms were sexual problem (31.20%); cardiac discomfort (19.10%) and bladder problem (12.80%).

**Conclusions:**

The prevalence of menopausal symptoms found in this study correspond to other studies on Asian women however the prevalence of classical menopausal symptoms of hot flushes and sweating were lower compared to studies on Caucasian women.

## Background

Modern medicine has significantly prolonged human lifespan [1]. All women who live long enough will make transition to menopause [2]. Menopause which is defined as complete cessation of menstruation for twelve months or more is a normal physiological change experienced by middle age women. Some of menopausal symptoms experienced by these women can be severe enough to affect their normal lifestyle. Unfortunately majority of these women are not aware of the changes brought about by menopause [3-5]. The common climacteric symptoms experienced by them can be group into: vasomotor, physical, psychological or sexual complaints. It was also noted in some postmenopausal women with long term estrogen deficiency, changes to the cardiovascular or bone which leads to osteoporosis. It is well documented that menopausal symptoms experienced by women affect their quality of life [6]. In Western countries, menopausal symptoms such as hot flushes, sweating and vaginal dryness are considered as the main climacteric complaints. In other cultures, these symptoms dramatically vary from those observed in Western women [7], while North American and European samples were reported higher rates of symptoms than that of Asian women [8,9]. It has been suggested that Asian women suffer more from the atypical symptoms and fewer, and with lesser severity, the typical psychological and vasomotor symptoms in comparison to those reported in Caucasian women in the west [6,7]. These symptoms are directly resulted from depletion of estrogen level as women approaches menopausal stage and some of these women begin to experiences these menopausal symptoms early in the perimenopausal phase [3].

Studies show that perimenopausal and postmenopausal women have more menopausal complaints than that of premenopausal women. They were noted to complain significantly more of vasomotor, sexual and psychological symptoms compared to premenopausal women [10,11].

Although menopause related symptoms have been extensively studied in the western countries, few data are available in Asia especially in South East Asia [3] including Bangladesh. This study was conducted in Kushtia, which is located in the western part of Bangladesh. This study aimed to document the menopausal-related symptoms among middle age women of Kushtia region of Bangladesh.

## Methods

### Participants and Setting

This is a cross-sectional study. Conducted from the month of April 2010 to August 2010. This study was approved by University of Development Alternative Research and Ethics Committee. The questionnaire based interview was carried out among women ages of 40 to 70 years who visited the government and private healthcare centers in Kushtia, Bangladesh. Five hundred and sixty participants were approached among them five hundred and nine participants take part in the study. The inclusion criteria consisted of women between the ages of 40 to 70 years who gave written consent to participate in this study. Pregnant and breast feeding women, women with uncontrolled medical conditions such as hypertension, diabetes mellitus or heart disease, who were undergoing treatment for serious diseases like cancer, women who were in remission, who had history of drug or alcohol abuse and who were on hormone replacement therapy were excluded from the study.

### Study Questionnaire

The questionnaire used in this survey was based on the Menopause Rating Scale (MRS) [3,9]. Questionnaire was modified to suit the Bangladeshi culture and norms. These changes allowed the researchers to cover all the profession groups in the female population living in the city, such as housewives, workers, teachers, engineers, adjudicators etc. Reliability analysis was performed on the modified Menopause Rating Scale questionnaires with Cronbach's alpha of somatic subscale 0.712, psychological subscale 0.743 and urogenital subscale 0.821. Therefore, this study determined the prevalence of menopausal symptoms and not the severity of the symptoms.

Questionnaires were divided into three sections:

(1) Socio-demographic data of the participants, which included: age, race, religion, marital status, educational level, occupation and average household income.

(2) Menopausal status of the participants: The menopausal status was classified according to STRAW (Stages of Reproductive Aging Workshop) classification which divided menopause staging into: *Postmenopausal*; no menstrual bleeding in the previous/last 12 months. *Late perimenopause*; had menstruation in the previous/last 2-12 months but not in the previous/last 2 months. *Early perimenopause*; had increasing irregularity of menses without skipping periods (7 days difference from the beginning of a given cycle to the next, experienced after the previously regular cycle) and *Premenopause*; minor changes in cycle length particularly decreasing length of the cycle. To aid statistical analysis, the *early *and *late perimenopausal *transition stages were grouped together as *perimenopausal stage*.

(3) Menopause Rating Scale (MRS) questionnaire were used as a basis for assessing menopausal symptoms in this study, this is a self-administered instrument which has been widely used and validated and have been used in many clinical and epidemiological studies, and in research on the etiology of menopausal symptoms to assess the severity of menopausal symptoms [12].

The questionnaire was first translated into Bengali language by a group of experienced healthcare workers and language experts, and then translated back to English to validate that the original meaning of the questionnaire was maintained in the translation. Moreover, a pilot study was done on 30 women to validate the translated questionnaires. The women were asked whether or not they had experienced the menopausal symptoms shown in the questioner in the previous one month (30 days). However, it was noted from the pilot study, these women had difficulties in rating the scale themselves, in order to minimize these difficulties, a face-to-face interviewed was done rather than using self-administered respond.

All women were interviewed in Bengali language. Face to face interviews were done on all the women by trained healthcare personnel who had undergone training, as this was important to make sure right answer were given and explanations can be given if the women were in doubt or unclear about the questions asked. At the reception desk during registration, women who fulfill the criteria were invited to participate in this study. Explanations were given and written informed consent was taken from them.

### Statistical Analysis

The Statistical Package for the Social Sciences software Version 14.0 (SPSS, Chicago, IL) was used for univariate analyses. The *X*^2 ^test was applied to compare the frequencies of the symptoms among the different menopausal status. The level P < 0.05 was considered as the cut-off value for significance.

## Results

Five hundred and nine women took part in the study. The mean age of respondents in this study was 54.50 ± 5.70 (SD) years. And the mean age at menopause was 51.14 ± 2.11 (SD) years. Among these women, 23.96% were premenopausal, 42.43% perimenopausal and 33.59% postmenopausal. Most of the women studied were Muslim (90.56%), some were Hindu (8.64%) and very few were from other Religions (0.78%). On the contrary, the percentage of widowed-divorced was much lower (21.41%) than that of married women (77.40%), but very few of them were unmarried (1.17%). In addition, the proportion of women who received nonacademic education was the highest (48.33%), whereas the lowest proportion of women was highly educated (5.5%). However, 85.06% of the women were housewives and 14.93% of women were involved with paid work. The highest percentage of women were from families with average income, whereas lowest were from families with high income and figures were 66.29% and 14.14% respectively. Moreover, the proportion of women who do regular physical exercise were much lower (21.02%) than that of who do not do any kind of exercise (44.40%). The lion share of the studied women claimed their health as average (50.09%) and a much lower percentage of women rated their health poor or very poor (11.98%).

Table 1 shows the detail frequency of menopausal symptoms. The five most common menopausal symptoms for all women (n = 509) were: Feeling tired (92.90%), headache (88.80%), joint and muscular discomfort (76.20%), physical and mental exhaustion (60.90%) and sleeplessness (54.40%). Table 2 shows the percentage of menopausal symptoms on individual menopausal status.

**Table 1:** Frequency of menopausal symptoms among Bangladeshi women aged 40-70 years in Kushtia,

Symptoms	n = 509	100%
1. Dryness of vagina	183	36
2. Joint and muscular discomfort	388	76.2
3. Physical and mental exhaustion	310	60.9
4. Sleeping problems	277	54.4
5. Hot flushes, sweating	182	35.8
6. Irritability	183	36
7. Anxiety	174	34.2
8. Depressive mood	190	37.3
9. Sexual problems	159	31.2
10. Heart discomfort	97	19.1
11. Bladder problems	65	12.8
12. Headache	452	88.8
13. Feeling tired	473	92.9

**Table 2:** Percentage of menopausal symptoms in the participants according to menopausal status

Menopausal symptoms	All	Premenopausal	Perimenopausal	Postmenopausal
Dryness of vagina	36	11.4	43.9*	43.2**^‡^
Joint and muscular discomfort	76.2	35.2	88.88*	86.4‡
Physical and mental exhaustion	60.9	40.1	69.44	64.9‡
Sleeping problems	54.4	29.5	58.7*†	52.6
Hot flushes, sweating	35.8*	27.8	37.5	39.1
Irritability	36	29.5	38.8*†	36.8‡†
Anxiety	34.2	25.4	35.6†	38.59
Depressive mood	37.3	30.3	35.6	44.4‡
Sexual problems	31.2	18.8	35.1†	35‡
Heart discomfort	19.1	11.4	25.9*†	15.7
Bladder problems	12.8	5.7	14.8	15.2**‡
Headache	88.8	77.86	91.2†	93.5
Feeling tired	92.9	88.5	94.4	94.1

* Significant difference p < 0.05 compared to premenopausal.

† Significant difference p < 0.05 compared to postmenopausal.

‡ Significant difference p < 0.05 compared to premenopausal.

** Significant difference p < 0.05 compared to perimenopausal.

## Discussion and Conclusion

The age of natural menopause has been examined in several North American studies and compared to that reported from other regions of the world [13]. The mean age at menopause in this study was 51.14 ± 2.11 years. This is slightly higher than studies done in Singapore (49.1 years), and Thailand (48.7 years) [14,15]. However, comparing with other research our findings still falls between the normal ranges of menopausal age [6,13,16,17].

In our study, the classical presentation of menopausal symptoms; feeling tired (92.9%) and headache (88.8%) which is quite similar when compare to other study [9]. The other classical presentation of menopausal symptoms; hot flushes, sweating and night sweats were noted to be lower (35.8%) in comparison to findings from studies done on western women which were reported to be from 45% to 75%. However, our findings of low menopausal classical symptoms were shared by studies done in other Asian countries [6,17].

From our study, joints and muscular discomfort; physical and mental exhaustions and sleeping problems (Table 2) were experienced most by perimenopausal followed by postmenopausal women and these were also statistical significant differences in comparison to premenopausal women. These findings were also noted to be corresponding to studies conducted among Asian and Caucasian women [3,18-21]. Frequency of sexual problems, bladder problems and vaginal dryness were experienced mainly by perimenopausal and postmenopausal group of women and it was also significant statistically in comparison to other menopausal status and similar finding were documented from other studies [14,6,18,21].

There are some limitations of this study. As this was a cross sectional study, it does not exclude other confounding effects of the natural aging process that may influence experience of symptoms. This study used modified MRS questionnaire, translated to Bengali, so there is a question of accuracy in translation, though this was done by a group of healthcare-workers and language experts. Although MRS is a self reporting questionnaire and substantial number of women studied does not have formal education, thus in orders to include these illiterate women, interviews were used instead. Moreover, some subjects could have been misclassified into the incorrect menopause status group.

## Consent

The study was performed with informed consent and following all the guidelines for experimental investigations required by the Institutional Research and Ethics Committee.

## Competing interests

The authors declare that they have no competing interests.

## Authors' contributions

SR participated in the design, coordinating and carried out the study and drafted the manuscript. FS and AI performed the statistical analysis and drafted the manuscript. All authors read and approved the final manuscript.
